# Promoting Self-management and Patient Activation Through eHealth: Protocol for a Systematic Literature Review and Meta-analysis

**DOI:** 10.2196/38758

**Published:** 2023-03-02

**Authors:** Saeed Moradian, Roma Maguire, Geoffrey Liu, Monika K Krzyzanowska, Marcus Butler, Chantal Cheung, Marisa Signorile, Nancy Gregorio, Shiva Ghasemi, Doris Howell

**Affiliations:** 1 School of Nursing Faculty of Health York University Toronto, ON Canada; 2 University of Strathclyde Glasgow United Kingdom; 3 Princess Margaret Cancer Centre Toronto, ON Canada; 4 University of Toronto Toronto, ON Canada; 5 Western University London, ON Canada; 6 Shahid-Beheshti University of Medical Sciences Tehran Iran

**Keywords:** digital health, cancer supportive care, cancer-related symptoms, self-efficacy, supported self-management

## Abstract

**Background:**

Major advances in different cancer treatment modalities have been made, and people are now living longer with cancer. However, patients with cancer experience a range of physical and psychological symptoms during and beyond cancer treatment. New models of care are needed to combat this rising challenge. A growing body of evidence supports the effectiveness of eHealth interventions in the delivery of supportive care to people living with the complexities of chronic health conditions. However, reviews on the effects of eHealth interventions are scarce in the field of cancer-supportive care, particularly for interventions with the aim of empowering patients to manage cancer treatment–related symptoms. For this reason, this protocol has been developed to guide a systematic review and meta-analysis to assess the effectiveness of eHealth interventions for supporting patients with cancer in managing cancer-related symptoms.

**Objective:**

This systematic review with meta-analysis is conducted with the aim of identifying eHealth-based self-management intervention studies for adult patients with cancer and evaluating the efficacy of eHealth-based self-management tools and platforms in order to synthesize the empirical evidence on self-management and patient activation through eHealth.

**Methods:**

A systematic review with meta-analysis and methodological critique of randomized controlled trials is conducted following Cochrane Collaboration methods. Multiple data sources are used to identify all potential research sources for inclusion in the systematic review: (1) electronic databases such as MEDLINE, (2) forward reference searching, and (3) gray literature. The PRISMA (Preferred Reporting Items for Systematic Reviews and Meta-Analyses) guidelines for conducting the review were followed. The PICOS (Population, Interventions, Comparators, Outcomes, and Study Design) framework is used to identify relevant studies.

**Results:**

The literature search yielded 10,202 publications. The title and abstract screening were completed in May 2022. Data will be summarized, and if possible, meta-analyses will be performed. It is expected to finalize this review by Winter 2023.

**Conclusions:**

The results of this systematic review will provide the latest data on leveraging eHealth interventions and offering effective and sustainable eHealth care, both of which have the potential to improve quality and efficiency in cancer-related symptoms.

**Trial Registration:**

PROSPERO 325582; https://www.crd.york.ac.uk/prospero/display_record.php?RecordID=325582

**International Registered Report Identifier (IRRID):**

DERR1-10.2196/38758

## Introduction

The incidence of cancer is rising, and it is estimated that by 2040, globally, more than 28 million people will experience cancer as new cancer cases [1]. It is expected that nearly half of Canadians will develop cancer in their lifetimes [2]. The main goal of a cancer treatment program is to cure or considerably prolong the life of patients and to ensure the best possible quality of life for cancer survivors [3]. Major advances in different cancer treatment modalities (ie, surgery, chemotherapy, radiotherapy, hormonal therapy, and biological response modifiers) have been made, and people are now living longer with cancer than they were in the past [3-5]. However, patients with cancer experience from a range of physical and psychological symptoms during their cancer journey. These symptoms are either directly related to the adverse effects of cancer or arise from the different types of treatments and may range from mild and temporary to severe, chronic, and life-threatening [6]. Moreover, symptoms impact daily physical function and can lead to or exacerbate psychological distress and worse health-related quality of life [7,8].

Globally, there is recognition that patients benefit from being actively engaged in their own health [9]. Active engagement of patients is considered critical to minimize the consequences of disease in daily living, support a better quality of life [10], and reduce health care costs [9].

eHealth interventions could potentially enhance the clinical, organizational, and relational aspects of care by integrating patient databases for individualized treatment and real-time decision support. Moreover, it has been reported that electronic technology, by identifying decision support, care coordination, and continuity of care, could improve cancer care delivery [11]. This approach can empower patients to manage their symptoms, improve patient-professional interactions, prevent unplanned hospital admissions, and reduce health care costs [12,13]. Additionally, for nurses, working with innovations such as mobile health (mHealth) in practice is becoming essential as it may facilitate the provision of quality care [14].

Even though there is empirical evidence that substantiates the role of eHealth interventions in the delivery of supportive care to people living with the complexities of chronic health conditions [15-19], the effects of eHealth interventions specifically designed for supporting patients with cancer to manage cancer-related symptoms and the effects on outcomes, that is, symptom burden, are less clear [20,21].

Reviews on the effects of eHealth interventions are scarce in the field of cancer supportive care, particularly for interventions to increase patient activation and empower patients to self-manage cancer-related symptoms. There is substantial evidence that patients who have the appropriate information and skills are more likely to engage actively in their care and effectively manage the consequences of treatment, including their physical and psychological symptoms [13,22]. Furthermore, information on how these interventions were planned and carried out and who benefited from these approaches is still required [23].

We propose to conduct a systematic review with meta-analysis and methodological critique of the literature to answer the following PICO (Population, Intervention, Comparison, Outcomes) research question: What is the efficacy in cancer populations (population: any phase of cancer, treatment, survivorship, palliative, and end of life care) of eHealth interventions (intervention) compared to usual care or other active intervention (comparison) on symptom severity, psychological distress, self-management behaviors, health outcomes, and health use (emergency department use, unplanned visits to the health care provider, hospitalization, patient activation, and patient empowerment; outcomes).

This review aims to explore usage and effectiveness of eHealth interventions designed to support patients with cancer in managing cancer-related symptoms and the effects on outcomes. The findings could inform and promote evidence-informed oncology practice for eHealth interventions targeted at cancer and advance science in the field.

## Methods

### Overview

**Figure 1:**
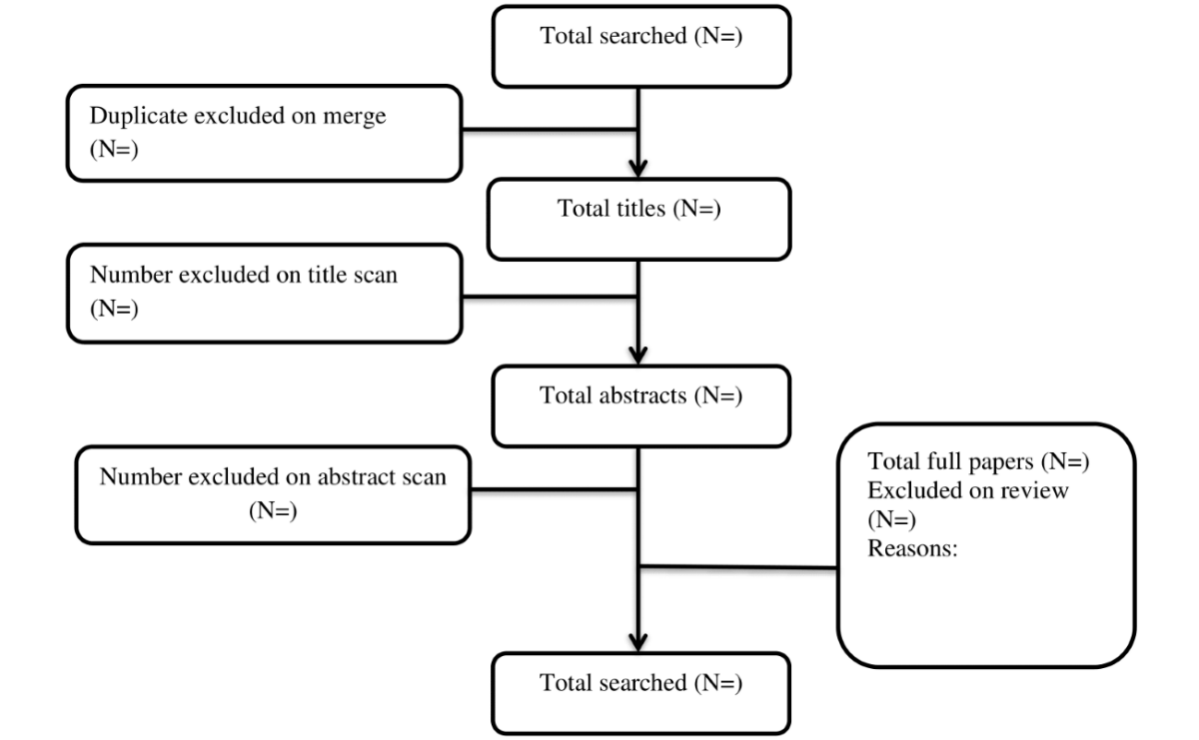
CONSORT (Consolidated Standards of Reporting Trials) chart: self-management and patient activation through eHealth review.

This systematic review to identify randomized controlled trials (RCTs) and meta-analyses follows methods as specified by the Cochrane Collaboration [24] and the Preferred Reporting Items for Systematic Reviews and Meta-Analyses guidelines (see Figure 1) [25]. A broad search to identify trial evidence for eHealth interventions to empower cancer patients to manage their symptoms, increase patient activation, and improve patient-professional interactions will be conducted. We posed the following specific research questions.

### Primary Research Question

Are eHealth interventions (or programs) effective in reducing the physical or psychological effects of cancer and its treatment or improving other health outcomes (ie, function, health-related quality of life, health use, or costs) compared with usual care or other active treatment?

### Secondary Research Questions

Does effectiveness (effect sizes [ESs]) of eHealth interventions (or programs) differ by patient or disease characteristics (age, race or ethnicity, education, cancer type, stage or phase in trajectory, treatment modality, or other antecedent personality variables such as optimism or trait anxiety), that is, effect modifiers?

Does effectiveness (ESs) of eHealth interventions (or programs) differ based on intervention design (delivery setting) or methods (eHealth-based self-management tools and platforms), training and qualities of the interventionist, intervention components, length of the intervention, or other potential mediators, that is, adherence to the intervention?

### Literature Search Strategy

The search strategy was developed with assistance from a library information specialist. A computerized search of electronic databases will be conducted from January 2000 until January 2022 as follows: The Cochrane Central Registry of Controlled Trials (CENTRAL), Cochrane Library and Trials Registry and the Database of Abstracts of Reviews of Effectiveness (DARE), MEDLINE (2000 to January 2022), Embase (2000 to January 2022), CINAHL (2000 to January 2022), PSYCHINFO (2000 to January 2022), and CancerLit (2000 to January 2022). In addition, the gray literature databases, EAGLE (2000 to January 2022), openSIGLE (2000 to January 2022), and PsychEXTRA (2000 to January 2022) will be searched. The reasons for limiting the literature search from 2000 onwards are the development of internet technology and the use of internet-based support programs in the delivery of supportive care over the past 22 years [26].

### Search Terms

The search for eligible studies will include search terms: self-manage* or “self-manage*” or self-car* or “self-care*”, behave* or cognitive* or train* or instruct* or patient education or “patient education” or “management plan*” or “management program* (AND) ehealth* or mHealth* or “mobile Health*” or Telehealth* (AND) terms for neoplasms, cancers, or cancer symptoms (fatigue, nausea or vomiting, pain, depression, anxiety, insomnia) and sensitive search terms for identifying randomized trials as subject headings specified by Cochrane [27]. The initial search strategy will be developed in MEDLINE (Multimedia Appendix 1) and will be adapted for all other databases.

### Types of Studies

Eligible studies will be identified based on the inclusion or exclusion criteria described below.

#### Inclusion Criteria

The following inclusion criteria were used:

- RCTs, quasi-randomized, or controlled clinical trials with an intervention group and a comparison patient group (receiving another intervention or standard care)
- Interventions using eHealth technology (web-based interventions, mHealth, etc)
- Interventions targeted at improving self-management or patient activation in managing physical symptoms or psychological distress
- Include one of the following outcomes: physical health status or symptoms, psychological health status or psychological symptoms or health distress, quality of life
- Comparisons were made with usual care or another active intervention
- Adults (18 years or older) at any stage or type of cancer

#### Exclusion Criteria

The following exclusion criteria were used:

- Studies without an eHealth intervention in the self-management of cancer
- Studies that did not include usual care controls or active intervention controls
- Studies published in non-English language
- Interventions that do not include a formal analysis of effects or use valid instruments

### Types of Outcome Measures

#### Primary Outcomes

We will be studying the following primary outcomes:

- Self-reported measures of physical health status or symptoms on valid and reliable scales
- Self-reported measures for psychological health status or symptoms, that is, depression or anxiety
- Quality of life or functioning is measured with valid and reliable scales

#### Secondary Outcomes

We will be studying the following secondary outcomes:

- Patient characteristics, including demographic and clinical data, and the intervention (intervention type, delivery, and intervention components)
- Participant’s withdrawal from the studies
- Subjective and objective measures of physical function or disability
- Symptom limitations or interference
- Healthcare utilization (hospital admissions, emergency department use, urgent visits to the family physician), and other health care or social costs

### Selection of Studies

Studies will be selected using Covidence software based on a review of the title, keywords, and abstract and coded using the following criteria: (1) include: an RCT, with a focus on cancer patient activation or self-management; (2) exclude: no self-management focus. Selecting studies includes these steps: (1) using a reference management software to merge search results and remove duplicates; (2) examine all titles initially to remove articles that are clearly not eHealth, followed by an abstract review (if there is any uncertainty, the abstract is included for a full text review); and (3) retrieve the full study reports and assess compliance with eligibility criteria independently by 2 reviewers. Agreement will be examined using interrater agreement (<75 nonagreement); disagreements will be resolved by consensus or in consultation with a third reviewer. Authors| will clarify study eligibility criteria or missing data results if necessary. Interventions with more than 1 article will also be retrieved and reviewed to complement the data abstraction and quality assessment of the study (Multimedia Appendix 2).

### Data Abstraction and Management

Data will be abstracted using a data abstraction form developed for the review based on Cochrane methods. Data abstraction is independently assessed by 2 reviewers with reliability of coding assessed by computing Kappa, or percentage agreement, for categorical data and the intraclass correlation for continuous data. If any aspect of the study design and conduct is unclear, the study authors will be contacted to complete data abstraction. Two other review authors will check a random sample of the abstractions. Disagreements will be resolved by discussion, with arbitration by a third author if necessary, following an independent review of the study report in question. The data abstraction form will be pretested on a minimum of 5 studies. The abstracted data will include categories as per Cochrane: (1) source and setting; (2) methods; (3) participants; (4) experimental interventions (extent to which specific intervention components delivered as described [adherence]; number, length, and frequency of implementation of intervention components; and characteristics of the interventionists); (5) control treatment; (6) analysis; (7) adverse events; (8) outcome measures; (9) results; (10) conclusions of study authors; and (11) miscellaneous, that is, funding sources.

### Assessment of Study Quality

A quality assessment will be performed by 2 review authors and checked by another author. A methodological quality assessment of studies will be conducted based on an adapted version of the Cochrane Collaboration Back Review Group criteria [28], which were previously used in other systematic reviews of internet-based interventions [29,30]. The Cochrane criteria was modified to better suit the type of examined studies: specification of eligibility criteria, randomized groups, treatment allocation concealed, groups similar at baseline, explicit description of interventions, description of compliance, description of dropout and comparison with completers, long-term follow-up (>3 months after postintervention assessment), timing of outcome assessment comparable, sample size described with power calculation, intention-to-treat analyses, and point estimates and measures of variability. The quality score could range from 0 to 12 points. For each study, all criteria will be scored as yes, no, or unclear, resulting in a maximum quality score of 12. In line with other researchers [29-31], studies obtaining at least two-thirds of the total score (ie, ≥8 points) will be considered high quality. Studies scoring 4 to 7 points will be rated as moderate quality, and studies scoring lower than 4 points will be rated as low quality.

Authors will be contacted with 2 reminders to complete missing data. Reviewers will be blinded to the authors of study reports. For each of these potential sources of bias, a judgment of yes (low risk of bias), no (high risk of bias), or unclear is assigned to each study (number of yeses is the single score or study). A summary table of the risk of bias across studies will be developed for reporting purposes.

### Data Analysis and ES Calculation

Outcomes will be analyzed as continuous or dichotomous variables depending on data reporting using standard statistical techniques. For continuous data (ie, symptom severity), a standardized mean difference with 95% CIs will be calculated as appropriate to facilitate comparison between intervention and controls with correction for differences in the direction of the scale. If reported as medians with ranges, means and SDs will be calculated [32]. For dichotomous outcomes, a relative risk ratio with 95% CIs is calculated.

#### Assessment of Heterogeneity

As per Cochrane, clinical heterogeneity (variability in the participants, interventions, and outcomes) [33,34] will be examined using the “I” squared statistic [35]. Random effects meta-analysis will be used if heterogeneity across studies cannot be explained; otherwise, a fixed-effects model will be used [33,36].

#### Subgroup Analysis

The following subgroup analysis will be conducted if the number of studies available for the analysis is adequate (10 studies for each characteristic modeled by participant characteristics and intervention components) as described below and based on the research questions posed:

1. Study intervention characteristics: The potential effect modifiers examined may include the characteristics of the intervention (length of follow-up, use of a number of components emphasized).
2. Study participant characteristics: Subgroup analysis will be conducted for disease stage (early and advanced), sex (male, female), and disease subtype, as some types of cancer may be associated with higher mortality.

#### Measurement of Treatment Effects and ES Calculation

A summary of findings table will be completed to synthesize the reporting of common primary outcomes: physical effects (function and symptoms), psychological effects (depression, anxiety, and health distress), and secondary outcomes (quality of life, health care use, and satisfaction) using the GRADEpro software. ES computations will be calculated using Hedges *g*. [37]. Where *g* cannot be computed directly from means and SDs based on the source paper, it will be computed indirectly from the available test statistics, for example, t, based on Rosenthal [38]. The estimates of *g* will be corrected for small-sample bias [37]. Given that outcomes could be differentially effective over different dimensions, particularly for symptoms, separate analyses for comparison, that is, physical symptoms (pain, fatigue, nausea or vomiting, insomnia), psychological symptoms (depression, anxiety, health distress), and separately for quality of life and use of health care services, will be conducted. For studies where a primary outcome is possible for the main analysis, this will require the identification of a primary outcome.

#### Sensitivity Analysis

A sensitivity analysis will be conducted to evaluate the robustness of the meta-analysis, that is, the effects of methodological quality on study outcomes, by assessing for associations between individual items in the methodological quality checklist and the study outcomes. When data can be pooled, sensitivity analysis will be conducted by pooling the “yes” versus “no” responses to risk. When the data cannot be pooled, the sensitivity analysis will be performed using a chi-square analysis as per Cochrane.

## Results

The literature search and data collection started in October 2017, after making a work plan to design and run the systematic search strategies in databases and the timeframe for delivery of the search results with the Library and Information Services within the University Health Network. However, to provide a comprehensive snapshot of knowledge since the time of incorporation of data from studies identified during the first search, a second literature search was conducted in February 2022 to ensure new studies were included and increase the validity of the review. The literature search yielded a total of 10,202 publications. Data will be summarized, and if possible, meta-analyses will be performed to evaluate the effectiveness of eHealth interventions on the outcomes. Results are expected to be published in winter 2023.

## Discussion

Recent literature has highlighted the utility of eHealth interventions with promising outcomes in cancer care, although mixed and inconclusive results were also presented [39]. The main contributions to this review will be the following: the use and effectiveness of eHealth interventions for supporting patients with cancer in managing cancer-related symptoms; identifying the key implications for better design, integration, and implementation that may have important effects on intervention outcomes; and a discussion, based on the data synthesized, on current gaps and limitations to inform better research toward all phases of development and evaluation of these interventions. Therefore, we will provide essential information for developing and implementing these interventions into clinical practice by providing recommendations based on the current best available evidence. The evaluation of the interventions might be limited by explicitly reporting the interventions. Any modifications or revisions made to the protocol will be presented in the final reports**.**

## Appendix

Multimedia Appendix 1 Ovid MEDLINE search history.

Multimedia Appendix 2 Data abstraction form.

## Abbreviations

ES: effect size
mHealth: mobile health
RCT: randomized controlled trial
