# Supplementary material for: Promoting Self-management and Patient Activation Through eHealth: Protocol for a Systematic Literature Review and Meta-analysis
Source: JMIR Res Protoc. 2023 Mar 2;12:e38758. doi: 10.2196/38758 (PMC10020897; doi:10.2196/38758)
Supplement: Multimedia Appendix 1 [file resprot_v12i1e38758_app1.docx]

**Multimedia Appendix 1**

Table S1 : Ovid MEDLINE(R) Search History

|  |  |  |  |  |  |  |
| --- | --- | --- | --- | --- | --- | --- |
| **#** | **Searches** | **Results** | **Type** |  |  |  |
|  | | | | | | |
| 1 | exp Neoplasms/ | 3163602 | Advanced |  |  |  |
| 2 | neoplas*.mp,kw. | 2720152 | Advanced |  |  |  |
| 3 | paraneoplas*.mp,kw. | 12167 | Advanced |  |  |  |
| 4 | cancer*.mp,kw. | 1428722 | Advanced |  |  |  |
| 5 | tumo?r*.mp,kw. | 1805251 | Advanced |  |  |  |
| 6 | onco*.mp,kw. | 449721 | Advanced |  |  |  |
| 7 | metast*.mp,kw. | 465603 | Advanced |  |  |  |
| 8 | malignan*.mp,kw. | 479558 | Advanced |  |  |  |
| 9 | carcin*.mp,kw. | 901581 | Advanced |  |  |  |
| 10 | adenocarc*.mp,kw. | 211230 | Advanced |  |  |  |
| 11 | lymphoma*.mp,kw. | 216645 | Advanced |  |  |  |
| 12 | leuk?emia*.mp,kw. | 292610 | Advanced |  |  |  |
| 13 | sarcoma*.mp,kw. | 109269 | Advanced |  |  |  |
| 14 | blastoma*.mp,kw. | 1171 | Advanced |  |  |  |
| 15 | melanoma*.mp,kw. | 114708 | Advanced |  |  |  |
| 16 | melanotic*.mp,kw. | 2886 | Advanced |  |  |  |
| 17 | neurilemmoma*.mp,kw. | 13442 | Advanced |  |  |  |
| 18 | nsclc.mp,kw. | 29622 | Advanced |  |  |  |
| 19 | osteosarcom*.mp,kw. | 26742 | Advanced |  |  |  |
| 20 | phyllodes.mp,kw. | 2101 | Advanced |  |  |  |
| 21 | cystosarcom*.mp,kw. | 614 | Advanced |  |  |  |
| 22 | fibroadenom*.mp,kw. | 3965 | Advanced |  |  |  |
| 23 | hepatoma*.mp,kw. | 26844 | Advanced |  |  |  |
| 24 | hepatoblastom*.mp,kw. | 3331 | Advanced |  |  |  |
| 25 | plasmacytoma*.mp,kw. | 10179 | Advanced |  |  |  |
| 26 | myeloma?.mp,kw. | 53394 | Advanced |  |  |  |
| 27 | lymphangioma*.mp,kw. | 6599 | Advanced |  |  |  |
| 28 | lymphangiomyoma*.mp,kw. | 491 | Advanced |  |  |  |
| 29 | lymphangiosarcoma*.mp,kw. | 462 | Advanced |  |  |  |
| 30 | lymphoblastoma*.mp,kw. | 350 | Advanced |  |  |  |
| 31 | lymphocytoma*.mp,kw. | 321 | Advanced |  |  |  |
| 32 | lymphosarcoma*.mp,kw. | 5055 | Advanced |  |  |  |
| 33 | immunocytoma*.mp,kw. | 486 | Advanced |  |  |  |
| 34 | angiosarcoma*.mp,kw. | 5313 | Advanced |  |  |  |
| 35 | astrocytoma*.mp,kw. | 20226 | Advanced |  |  |  |
| 36 | neuroma*.mp,kw. | 13429 | Advanced |  |  |  |
| 37 | cytoma?.mp,kw. | 42 | Advanced |  |  |  |
| 38 | gist.mp,kw. | 5198 | Advanced |  |  |  |
| 39 | neurocytoma?.mp,kw. | 772 | Advanced |  |  |  |
| 40 | hodgkin*.mp,kw. | 84548 | Advanced |  |  |  |
| 41 | non-hodgkin*.mp,kw. | 51518 | Advanced |  |  |  |
| 42 | nonhodgkin*.mp,kw. | 114 | Advanced |  |  |  |
| 43 | incidentaloma?.mp,kw. | 1691 | Advanced |  |  |  |
| 44 | retinoblastoma?.mp,kw. | 20605 | Advanced |  |  |  |
| 45 | plasmacytoma*.mp,kw. | 10179 | Advanced |  |  |  |
| 46 | cholangiocarcinoma*.mp,kw. | 11232 | Advanced |  |  |  |
| 47 | leiomyoblastoma*.mp,kw. | 391 | Advanced |  |  |  |
| 48 | leiomyosarcoma*.mp,kw. | 10933 | Advanced |  |  |  |
| 49 | melanosis.mp,kw. | 4053 | Advanced |  |  |  |
| 50 | (hutchinson* adj2 freckle*).mp,kw. | 666 | Advanced |  |  |  |
| 51 | melanoameloblastom*.mp,kw. | 11 | Advanced |  |  |  |
| 52 | melanoblastom*.mp,kw. | 490 | Advanced |  |  |  |
| 53 | melanocarcin*.mp,kw. | 101 | Advanced |  |  |  |
| 54 | melanomalign*.mp,kw. | 48 | Advanced |  |  |  |
| 55 | naevocarcin*.mp,kw. | 34 | Advanced |  |  |  |
| 56 | nevocarcin*.mp,kw. | 69 | Advanced |  |  |  |
| 57 | ameloblastom*.mp,kw. | 4033 | Advanced |  |  |  |
| 58 | adenosquam*.mp,kw. | 3291 | Advanced |  |  |  |
| 59 | adamantinom*.mp,kw. | 947 | Advanced |  |  |  |
| 60 | teratoma*.mp,kw. | 20615 | Advanced |  |  |  |
| 61 | metaplas*.mp,kw. | 22202 | Advanced |  |  |  |
| 62 | rhabdomyosarcoma?.mp,kw. | 13266 | Advanced |  |  |  |
| 63 | liposarcoma?.mp,kw. | 6386 | Advanced |  |  |  |
| 64 | myxofibrosarcoma?.mp,kw. | 401 | Advanced |  |  |  |
| 65 | neurofibrosarcoma?.mp,kw. | 479 | Advanced |  |  |  |
| 66 | chondrosarcoma?.mp,kw. | 8647 | Advanced |  |  |  |
| 67 | hemangiopericytoma?.mp,kw. | 3432 | Advanced |  |  |  |
| 68 | hemangioendothelioma?.mp,kw. | 4055 | Advanced |  |  |  |
| 69 | neuroblastoma?.mp,kw. | 38054 | Advanced |  |  |  |
| 70 | or/1-69 | 4108474 | Advanced |  |  |  |
| 71 | Self-Management/ | 1102 | Advanced |  |  |  |
| 72 | exp Self Care/ | 51764 | Advanced |  |  |  |
| 73 | Self Administration/ | 10889 | Advanced |  |  |  |
| 74 | Self Medication/ | 4550 | Advanced |  |  |  |
| 75 | self efficacy/ | 18401 | Advanced |  |  |  |
| 76 | Self-Help Groups/ | 8804 | Advanced |  |  |  |
| 77 | Patient Education as Topic/ | 81885 | Advanced |  |  |  |
| 78 | Patient Participation/ | 23770 | Advanced |  |  |  |
| 79 | "Power (Psychology)"/ | 12260 | Advanced |  |  |  |
| 80 | exp Cognitive Therapy/ | 25541 | Advanced |  |  |  |
| 81 | self car*.mp,kw. | 38564 | Advanced |  |  |  |
| 82 | selfcar*.mp,kw. | 96 | Advanced |  |  |  |
| 83 | self manag*.mp,kw. | 14090 | Advanced |  |  |  |
| 84 | selfmanag*.mp,kw. | 27 | Advanced |  |  |  |
| 85 | self administ*.mp,kw. | 40309 | Advanced |  |  |  |
| 86 | selfadminist*.mp,kw. | 51 | Advanced |  |  |  |
| 87 | self medicat*.mp,kw. | 6579 | Advanced |  |  |  |
| 88 | selfmedicat*.mp,kw. | 13 | Advanced |  |  |  |
| 89 | self monitor*.mp,kw. | 10552 | Advanced |  |  |  |
| 90 | selfmonitor*.mp,kw. | 15 | Advanced |  |  |  |
| 91 | self efficac*.mp,kw. | 27547 | Advanced |  |  |  |
| 92 | selfefficac*.mp,kw. | 20 | Advanced |  |  |  |
| 93 | self guid*.mp,kw. | 423 | Advanced |  |  |  |
| 94 | self help*.mp,kw. | 17289 | Advanced |  |  |  |
| 95 | selfhelp*.mp,kw. | 14 | Advanced |  |  |  |
| 96 | self regulat*.mp,kw. | 8938 | Advanced |  |  |  |
| 97 | selfregulat*.mp,kw. | 25 | Advanced |  |  |  |
| 98 | self direct*.mp,kw. | 4240 | Advanced |  |  |  |
| 99 | selfdirect*.mp,kw. | 9 | Advanced |  |  |  |
| 100 | self govern*.mp,kw. | 489 | Advanced |  |  |  |
| 101 | selfgovern*.mp,kw. | 1 | Advanced |  |  |  |
| 102 | self determin*.mp,kw. | 4092 | Advanced |  |  |  |
| 103 | selfdetermin*.mp,kw. | 3 | Advanced |  |  |  |
| 104 | cognit*.mp,kw. | 341744 | Advanced |  |  |  |
| 105 | (personal adj2 manag*).mp,kw. | 627 | Advanced |  |  |  |
| 106 | (symptom? adj3 manag*).mp,kw. | 9498 | Advanced |  |  |  |
| 107 | (symptom? adj3 monitor*).mp,kw. | 2105 | Advanced |  |  |  |
| 108 | ((patient? or client?) adj2 educat*).mp,kw. | 100454 | Advanced |  |  |  |
| 109 | ((patient? or client?) adj2 participat*).mp,kw. | 38373 | Advanced |  |  |  |
| 110 | ((patient? or client?) adj2 empower*).mp,kw. | 2483 | Advanced |  |  |  |
| 111 | ((patient? or client?) adj2 activation*).mp,kw. | 3368 | Advanced |  |  |  |
| 112 | (care adj1 recipient?).mp,kw. | 1567 | Advanced |  |  |  |
| 113 | or/71-112 | 621581 | Advanced |  |  |  |
| 114 | Internet/ | 68048 | Advanced |  |  |  |
| 115 | exp Online Systems/ | 15251 | Advanced |  |  |  |
| 116 | Computer-Assisted Instruction/ | 11474 | Advanced |  |  |  |
| 117 | User-Computer Interface/ | 35537 | Advanced |  |  |  |
| 118 | Mobile Applications/ | 4019 | Advanced |  |  |  |
| 119 | Precision Medicine/ | 14380 | Advanced |  |  |  |
| 120 | Telemedicine/ | 19300 | Advanced |  |  |  |
| 121 | internet.mp,kw. | 85287 | Advanced |  |  |  |
| 122 | online.mp,kw. | 74187 | Advanced |  |  |  |
| 123 | on-line.mp,kw. | 21159 | Advanced |  |  |  |
| 124 | web based*.mp,kw. | 21480 | Advanced |  |  |  |
| 125 | webbased*.mp,kw. | 21 | Advanced |  |  |  |
| 126 | web site?.mp,kw. | 6727 | Advanced |  |  |  |
| 127 | website?.mp,kw. | 17624 | Advanced |  |  |  |
| 128 | web page?.mp,kw. | 1428 | Advanced |  |  |  |
| 129 | webpage?.mp,kw. | 446 | Advanced |  |  |  |
| 130 | (computer* assist* adj3 instruct*).mp,kw. | 11594 | Advanced |  |  |  |
| 131 | (computer* adj3 self-instruct*).mp,kw. | 18 | Advanced |  |  |  |
| 132 | (user-computer* adj2 interface?).mp,kw. | 35578 | Advanced |  |  |  |
| 133 | (mobile adj2 app*).mp,kw. | 5537 | Advanced |  |  |  |
| 134 | (precision adj2 medicine).mp,kw. | 16325 | Advanced |  |  |  |
| 135 | telemed*.mp,kw. | 21740 | Advanced |  |  |  |
| 136 | tele-med*.mp,kw. | 113 | Advanced |  |  |  |
| 137 | telehealth*.mp,kw. | 3093 | Advanced |  |  |  |
| 138 | tele-health*.mp. | 106 | Advanced |  |  |  |
| 139 | ehealth*.mp,kw. | 1890 | Advanced |  |  |  |
| 140 | e-health*.mp,kw. | 1979 | Advanced |  |  |  |
| 141 | mhealth*.mp,kw. | 1616 | Advanced |  |  |  |
| 142 | m-health*.mp,kw. | 324 | Advanced |  |  |  |
| 143 | (mobile adj health*).mp,kw. | 5230 | Advanced |  |  |  |
| 144 | technolog* communic*.mp,kw. | 77 | Advanced |  |  |  |
| 145 | or/114-144 | 261854 | Advanced |  |  |  |
| 146 | 70 and 113 and 145 | 2681 | Advanced |  |  |  |
| 147 | exp animals/ not (exp animals/ and exp humans/) | 4576104 | Advanced |  |  |  |
| 148 | 146 not 147 | 2680 | Advanced |  |  |  |
| 149 | limit 148 to "all child (0 to 18 years)" | 361 | Advanced |  |  |  |
| 150 | limit 148 to "all adult (19 plus years)" | 1527 | Advanced |  |  |  |
| 151 | 149 not 150 | 80 | Advanced |  |  |  |
| 152 | 148 not 151 | 2600 | Advanced |  |  |  |
| 153 | limit 152 to yr="2017 -Current" | 500 | Advanced |  |  |  |
| 154 | limit 153 to english language | 491 | Advanced |  |  |  |
|  | | |  |  |  |  |
