# Supplementary material for: Promoting Self-management and Patient Activation Through eHealth: Protocol for a Systematic Literature Review and Meta-analysis
Source: JMIR Res Protoc. 2023 Mar 2;12:e38758. doi: 10.2196/38758 (PMC10020897; doi:10.2196/38758)
Supplement: Multimedia Appendix 2 [file resprot_v12i1e38758_app2.docx]

**Appendix 2: Data Abstraction Form**

**SELF-MANAGEMENT E-HEALTH INTERVENTIONS IN CANCER**

**DATA ABSTRACTION FORM**

|  | | | | | |
| --- | --- | --- | --- | --- | --- |
| **IDENTIFICATION FEATURES FOR THE STUDY** | | | | | |
| Unique Identifier | | |  | | |
| Author(s) | | |  | | |
| Title of Article | | |  | | |
| Author Contact Details and Institution | | |  | | |
| Clinical Trials Registry IdentIfier | | |  | | |
| Source | | |  | | |
| Country of Origin | | |  | | |
| Reviewer | | |  | | |
| Date of Study | | |  | | |
| Notes: | | | | | |
| **ELIGIBILITY** | | | | | |
| Study Characteristics (correct population, interventions, outcome, and study design) | | | | | |
| Confirmed | | | YES | | |
|  |  |  | **NO** | | |
| **If NO, Why excluded:** | | |  | | |
| **POPULATION CHARACTERISTICS/SETTING** | | | | | |
| Target Population | | |  | | |
| (describe, type of cancer) | | |  | | |
| Inclusion Criteria | | |  | | |
| **Total number of participants in each condition** | | | | | |
| Condition A | | |  | | |
| Condition B | | |  | | |
| Condition C | | |  | | |
| Condition D | | |  | | |
| Were intervention groups comparable? | | |  | | |
| **Characteristics of participants at Intervention Commencement (Baseline)** | | | | | |
| Age | | |  | | |
| Ethnicity | | |  | | |
| Socioeconomic | | |  | | |
| (income and/or education) | | |  | | |
| Sex | | |  | | |
| Geographical Region | | |  | | |
| Marital Status | | |  | | |
| Comorbid illness | | |  | | |
| Treatment | | |  | | |
| Other | | |  | | |
| Sample Calculation | | |  | | |
| Recruitment Procedures | | |  | | |
| **METHODS AND METHODOLOGICAL QUALITY (RISK OF BIAS)** | | | | | |
| Study Design | | |  | | |
| RCT | | |  | | |
| Type of Control | | |  | | |
| Total Study Duration | | |  | | |
| Full Description of Randomization/Sequence Generation | | |  | | |
| Details of Sequence Generation | | |  | | |
| Was the allocation sequence adequately generated? | | | **YES**  **NO** | | |
| Unit of Allocation | | |  | | |
| Concealment Method | | |  | | |
| Was allocation adequately concealed? | | |  | | |
| Quality Assessment | | |  | | |
| Blinding (participants, personnel, outcome assessors) | | |  | | |
| Was knowledge of the allocated intervention adequately prevented during the study? | | |  | | |
| Other Concerns about Bias | | |  | | |
| **INTERVENTIONS** | | | | | |
| Focus on intervention | | |  | | |
| Name of program/intervention: | | |  | | |
| Total number of intervention groups | | |  | | |
| (includes controls) | | |  | | |
| Details of intervention for each group for | | |  | | |
| each condition | | |  | | |
| Condition A | | |  | | |
| Condition B | | |  | | |
| **OUTCOMES AND OUTCOME MEASURES** | | | | | |
| What was measured at baseline? | | |  | | |
| What was measured after the intervention? | | |  | | |
| Who carried out the measurement? | | |  | | |
| Time intervals between first and second measurement? | | |  | | |
| Time intervals between first and last measurement? | | |  | | |
| Were incomplete outcome data adequately addressed? | | |  | | |
| Is the study free of suggestion of selective  outcome reporting? | | |  | | |
| **DETAILS OF SELF-MANAGEMENT COMPONENTS** | | | | | |
| Specify theoretical model | | |  | | |
| Structured Education Components | | |  | | |
| Core Skills Emphasis | | |  | | |
| Cognitive Symptom Management Skill  Training (emphasis on coping, managing  emotions/stress; therapeutic support;  relaxation therapy) | | |  | | |
| Goal setting/Action planning | | |  | | |
| Problem Solving Skills | | |  | | |
| Coaching in use of Support | | |  | | |
| Partnership/Communication/ with health  professionals | | |  | | |
| Enhancements for self-efficacy: i.e. teaching  for performance mastery; modeling; reinterpretation of symptoms; social  persuasion | | |  | | |
| **ANALYSIS** | | | | | |
| Statistical techniques used | | |  | | |
| Does technique adjust for confounding? | | |  | | |
| Handling of missing data | | |  | | |
| Procedures for intention to treat analysis | | |  | | |
| Numbers included in study design | | |  | | |
| Deviations from study design | | |  | | |
| Power calculation | | |  | | |
| Attrition rates | | |  | | |
| Number followed up from each condition | | |  | | |
| Condition A | | |  | | |
| Condition B | | |  | | |
| Attrition adequately dealt with? | | |  | | |
| Methods for additional analysis and  adjustments in analysis | | |  | | |
| Estimated effect size | | |  | | |
| **RESULTS** | | | | | |
| Variable Condition A | Variable Condition A | Variable Condition A | | Variable Condition A | Variable Condition A |
|  |  |  | |  |  |
|  |  |  | |  |  |
|  |  |  | |  |  |
|  |  |  | |  |  |
| Quantitative Results (eg. Estimates of effect size) | | |  | | |
| Cost of Intervention | | |  | | |
| Cost-effectiveness | | |  | | |
